# Examining the relationship between climate concern, climate anxiety and climate action in the UK

**DOI:** 10.1186/s40359-026-04170-9

**Published:** 2026-03-03

**Authors:** Emily Wolstenholme, Katharine Steentjes, Christina Demski, Wouter Poortinga

**Affiliations:** 1 School of Geography and Planning, Cardiff University, Cardiff, Wales, UK; 2 School of Psychology, Cardiff University, Cardiff, Wales, UK; 3 School of Psychology, Swansea University, Swansea, Wales, UK; 4 Department of Psychology, University of Bath, Bath, England, UK; 5 Welsh School of Architecture, Cardiff University, Cardiff, Wales, UK

**Keywords:** Climate concern, Climate anxiety, Climate action, Climate activism, Generational differences

## Abstract

The effects of climate change are being felt globally, resulting in increased extreme weather events and mental health challenges. Climate change concern and anxiety are widespread, especially among young people. Currently, little is known about how these responses to climate change may interact with each other to motivate or hinder different types of environmental action. This paper addresses this gap using two nationally representative surveys conducted in 2022 and 2023 in the UK (n_total_ = 2,087). The results show that individuals concerned about climate change were significantly more likely to have climate anxiety and that climate anxiety is subsequently significantly associated with an increased likelihood of engaging in pro-environmental action, including private-sphere behaviours (individual actions relating to diet, transport, household energy, travel, and material consumption) and climate activism (collective actions, including supporting protests and community initiatives). When controlling for the effect of climate anxiety, climate concern was still associated with an increased likelihood of engaging in pro-environmental action, although the effect was smaller. These results suggest that climate anxiety partly explains the effects of climate concern and is an additional driver of climate action. Additionally, the results show that climate anxiety was higher among younger respondents, and that the likelihood of engaging in pro-environmental actions decreases as age increases. However, the effects of climate change concern and anxiety on climate action were consistent across respondents of different ages, with no moderating effects of age. Other factors may be responsible for decreased climate engagement among older age groups demonstrated in past literature.

**Supplementary Information:**

The online version contains supplementary material available at 10.1186/s40359-026-04170-9.

## Background

In recent years, climate change has been recognised not only as an environmental crisis, but also as a significant mental health challenge. In addition to the physical impacts being felt from climate change, emotional responses such as worry, fear, and grief, are increasingly being felt. For example, the impacts of discrete events caused by climate change have been linked with increased levels of depression, anxiety, and trauma [1, 2]. On a national level, the UK has experienced record-breaking temperatures over the last decade [3], while concern about climate change has also steadily increased [4]. However, the negative impacts of climate change on mental health are not limited to those with direct experience of its effects. For example, climate anxiety can be experienced through mere perceptions of climate change and its impacts [5]. Recent studies have identified climate anxiety across different countries, with relatively low levels shown among adults [6–9]. Higher levels of climate anxiety have been shown among younger age groups [6, 7, 9], with climate change anxiety being widespread among young people across the world [10]. However, little is currently known about the extent to which different affective responses to climate change, including climate anxiety, may be adaptive in motivating climate action.

Climate concern and climate anxiety are related but distinct constructs. Climate concern is conceptualised mainly through cognitive factors, including risk perceptions and perceived importance of addressing climate change, in addition to an affective component, i.e. worry about climate change [11]. Climate anxiety conceptualises a greater level of psychological distress related to climate change [7]. The term climate anxiety has been used differently across various fields of research. For example, climate anxiety has been used to describe climate concern, worry, rumination, fear, shame, hopelessness, despair and guilt [5, 12, 13]. Other related concepts include eco-anxiety [14], ecological grief [15] and solastalgia [16]. Despite differences in conceptualisations of climate anxiety, it is commonly understood as a deeply held fear relating to environmental change and its impacts, specifically related to anthropogenic climate change [17]. The need for a reliable and consistent measure of climate anxiety was recently addressed through the validated Climate Change Anxiety Scale (CCAS; [7]). The CCAS characterises climate anxiety through cognitive-emotional impairment, including rumination and difficulty sleeping or concentrating, in addition to functional impairment, which interferes with a person’s ability to work or socialise [7]. This conceptualisation is more stringent than other definitions of climate anxiety [5] and succeeds in capturing clinically relevant levels of anxiety differentiated from general levels of climate change worry [7]. This distinction is relevant given that worry and anxiety may have differing impacts on individuals, potentially resulting in different levels of behavioural engagement.

According to Basic Emotion Theory, emotions allow individuals to respond to threats and opportunities in the environment [18]. Anxiety functions as a warning sign when survival or wellbeing is threatened [19]. As anxiety is future oriented, it can lead to adaptive preparations for future threat or danger [20, 21]. It is plausible therefore that climate anxiety may lead to relevant action towards mitigating rising greenhouse gas emissions and may even be necessary to motivate transitions towards a sustainable future [22]. Studies with adults from different age groups consistently show that concern about climate change is positively related to pro-environmental behaviours and intentions, including energy saving behaviours, sustainable food and travel choices, policy support and involvement in environmental organisations [23–26]. However, reviews of studies investigating the direct relationship between climate concern and different pro-environmental behaviours tend to show that the relationship is weak or moderate at best, often explaining not more than 10% of the variance in specific pro-environmental behaviours [27]. Moreover, research has shown that climate concern is mainly linked to pro-environmental behaviour when it is low cost and causes little inconvenience, with smaller effects for behaviours that are more costly or inconvenient [28–30]. Therefore, the extent to which climate concern leads to impactful pro-environmental behaviour may be limited.

While research on climate concern is widespread, studies investigating the link between climate anxiety and pro-environmental behaviour are more limited and have yielded mixed results. For example, Ogunbode et al. [31] found that climate anxiety was associated with pro-environmental behaviour in 25 countries and climate activism in 12 countries, out of a possible 32 surveyed countries [31]. Similarly, a national survey conducted in Finland showed climate anxiety was associated with climate action [32]. On the other hand, Clayton and Karazsia [7] found no relationship between climate anxiety and behavioural engagement across two studies conducted in the United States [7]. There is evidence that climate anxiety can be maladaptive and lead to avoidance behaviours [22, 33, 34]. For example, a study using Australian survey data showed the extent to which climate change made individuals feel anxious or afraid was associated with decreased collective climate action and disengagement with the pro-climate movement, indicating potential rebound effects [35]. This has led some authors to differentiate between constructive and unconstructive worry [11], in addition to motivating and ‘paralysing’ forms of anxiety [17, 36, 37]. However, it is also plausible that mixed findings regarding the effects of climate anxiety reflect inconsistencies in its conceptualisation and measurement across studies. More studies using a consistent, validated measure of climate anxiety, such as the CCAS, are needed to disentangle the effects of climate anxiety and climate concern or worry, on pro-environmental behaviour.

Considering the scale of lifestyle changes needed at an individual level to address climate change, it is important to consider determinants of pro-environmental action not limited to private-sphere behaviours [38]. Private-sphere behaviours refer to actions taking place in an individual’s everyday life that have a positive environmental impact, such as the use, purchase and disposal of goods [39, 40]. Unlike public-sphere behaviours, private-sphere behaviours do not require co-operation or collective action and typically have a relatively small impact on the environment [39, 40]. Stern (2000) differentiates environmentally significant behaviours further, into four main categories: private-sphere behaviours (e.g., recycling and green consumerism), non-activist public-sphere behaviours (e.g., policy support), activist public-sphere behaviours (e.g., involvement in environmental protests or demonstrations), and behaviours in organisations (e.g., decision-making and organisational practices) [39]. Although much of the literature has focussed on private-sphere behaviours, public-sphere behaviours and climate activism will play a critical role in achieving the transformative changes needed to address climate change [38, 41, 42].

While climate concern has predominantly been associated with private-sphere behaviours, the role of climate anxiety in motivating different types of pro-environmental behaviour remains unclear, although past literature has indicated that affective responses to climate change can motivate engagement with climate activism. For example, distress, encompassing worry and anxiety among other emotions, has been found to predict previous engagement with climate activism behaviours, including attending protests and rallies [43]. Moreover, Ogunbode et al. [31] found that climate anxiety measured using the State-Trait Anxiety Inventory [44] was positively associated with attendance to climate protests [31]. Climate-related emotions, including fear, have also been cited as reasons for engaging in climate activism [45]. Moreover, past research has shown individuals may be motivated to engage in climate activism as a means of turning their climate anxiety into something positive [46].

Affective engagement with climate change varies among different age groups, with younger generations showing greater affective engagement with the issue than older generations [47]. As climate change related events are becoming more common and will continue increase in the future, younger generations face more extreme changes in temperature and extreme weather events than older generations [1, 48]. It is therefore not surprising that young people experience greater levels of climate-related emotions, including fear, guilt and outrage in relation to climate change, in addition to higher levels of concern [49, 50] and climate anxiety [7, 9] compared to older age groups. Moreover, climate-related emotions worry, anger, and guilt, have significantly increased among younger generations in recent years, with smaller increases shown among older generations [51]. It is therefore important to consider how individuals of different ages may be differently impacted by climate anxiety and how this may subsequently influence behavioural engagement. For example, research has also shown a greater engagement with climate activism among young people relative to older age groups and among individuals experiencing more climate distress [43].

### The current study

Based on the above literature, this paper has two aims. First, it aims to investigate how climate change concern and climate anxiety may differently motivate two types of climate action, private-sphere behaviour and climate activism. Second, it aims to identify whether climate change concern and climate anxiety may have a stronger impact upon motivating climate action among younger individuals. This is done by analysing responses to a UK survey conducted in 2022 and 2023. The effects of climate change concern are observed with and without controlling for the effects of climate anxiety, to disentangle the effects of climate change concern and climate anxiety on motivating pro-environmental behaviour. Private-sphere behaviour and climate activism are investigated separately, to provide insight into whether climate concern and climate anxiety have a different role in motivating different types of climate action. Finally, potential moderating effects of age are investigated using interaction terms, to establish whether change concern and climate anxiety may have a stronger impact on motivating climate action among younger individuals.

## Methods

### The CAST survey

The data set was comprised from two waves of a national UK-based survey, collected in 2022 and 2023. In total, 2,087 respondents completed the surveys (*n* = 1,087 in 2022 and *n* = 1,000 in 2023). Data are missing where respondents did not respond to a question, responded with ‘*Don’t know’* or *‘prefer not to say’*. Respondents were aged between 18 and 98 years, with a mean age of 50 (SD = 16.90). Respondents identified as male (n = 942), female (n = 1,057), or other (n = 5). Level of education varied and included high-school or secondary school qualification (n = 985), undergraduate degree (n = 674), postgraduate degree (n = 272), and no formal qualifications (n = 75). A further breakdown of participant demographics can be viewed in Table 1.

**Table 1:** Respondent demographics

	*n*	%
**Age** ^1^		
18–24	164	8%
25–34	302	14%
35–44	438	21%
45–54	239	11 %
55–64	428	21%
65+	516	25%
Mean (SD)	50 (SD = 16.90)	
**Gender**		
Male	942	47%
Female	1057	53%
I prefer to describe my gender in another way	5	0%
Prefer not to say	5	0%
**Highest level of education**		
No formal qualifications	75	4%
High school or secondary school qualifications	985	48%
Undergraduate/college degree level (e.g. bachelor’s degree)	674	33%
Graduate/Postgraduate degree level (e.g. Masters, PhD)	272	13%
Other	37	2%
Prefer not to say	12	1%
**Ethnicity**		
Asian/Asian British	108	5%
Black/Black British	43	2%
Mixed (e.g. White & Asian, White & Black)	40	2%
White British	1672	83%
White Irish/White Other	134	7%
Other	15	1%
Prefer not to say	13	1%

^1^This grouping is for descriptive purposes only. Age was treated as a continuous variable in all analyses.

### Procedure

The two online surveys were commissioned by the Centre for Climate Change and Social Transformations (CAST) and facilitated by an external social research company, DJS Research. Data collection took place between 5th September and 6th October 2022 and 13th October and 14th November 2023. In return for their participation, respondents received compensation, including reward points that could be redeemed for cash and prizes or travel miles depending on the programme source. Quota sampling was used to ensure a nationally representative sample in terms of age, gender, region, and socio-economic status. Respondents reported sociodemographic information including age, education and gender, which were used in subsequent analysis. The full surveys included a range of questions on climate change beliefs and support for low carbon strategies and people’s attitudes and behaviours in relation to transport, diet, material consumption and thermal comfort.

### Measures

#### Climate concern

A climate concern scale was constructed using four items assessing worry “How worried, if at all, are you about climate change?” (from 1 not at all worried to 5 extremely worried), perceived threat “How serious a threat, if at all, is climate change to each of the following”: (1) “You and your family” (2), “The UK as a whole” (from 1 not at all serious to 5 extremely serious), and perceived urgency “Which of these best describes your views about the level of urgency with which climate change needs to be addressed?” (from 1 addressing climate change requires an extremely high level of urgency to 5 addressing climate change requires little or no urgency). This is based on past research using similar measures of climate concern [28, 50]. The scale had good internal consistency (Cronbach’s $\:\mathrm{a}$ = 0.89). Climate concern among respondents was moderate (M = 3.34, SD = 0.94).

#### Climate anxiety

The survey included a short version of the CCAS [7]. Six items were included following advice from the original scale developers, Clayton and Karazsia. Respondents were asked “Thinking about the following statements around climate change, how frequently if at all do the following apply to you?” (from 1 never to 5 almost always): “Thinking about climate change makes it difficult for me to concentrate”, “Thinking about climate change makes it difficult for me to sleep”, “I think ‘why can’t I handle climate change better?’”, “My concerns about climate change make it hard for me to have fun with my family or friends”, “My concerns about climate change interfere with my ability to get work or school assignments done” and “My concerns about climate change undermine my ability to work to my potential”. The resulting scale had excellent reliability (Cronbach’s $\:a$ = 0.95). The climate anxiety scale was transformed into a nominal variable given low average levels of anxiety among respondents (M = 1.74, SD = 0.89). Respondents were categorised into three groups, i.e (1), those who had no climate anxiety (averaged score of ‘never’ across all items) (2), those who had mild climate anxiety (averaged score of ‘rarely’ or below, but higher than ‘never’ across all items) and (3) those who had high climate anxiety (averaged score higher than ‘rarely’ across all items). In this study, 37% of respondents reported experiencing no climate anxiety at all, 49% reported mild levels of climate anxiety, and 14% reported high levels of climate anxiety.

#### Private-sphere behaviour

An overall measure of private-sphere behaviour was created from 13 items relating to different areas for potential climate mitigation: diet, transport, travel, household energy, and material consumption. Respondents were asked “Please indicate how likely or unlikely you are to take each of the following actions in the next 12 months?” (from 1 very unlikely to 5 very likely): “Eat fewer calories a day to reduce consumption”, “Plan meals ahead to avoid food waste”, “Follow a vegan diet”, “Follow a vegetarian diet”, “Buy locally produced food”, “Use a bike for commuting (or for other regular journeys)”, “Live car free”, “Go on holiday by train instead of flying”, “Buy an electric car”, “Keep your home at a colder temperature in the winter (by 1 degree)”, “Use leasing schemes instead of buying new (e.g. for washing machines, cars)”, “Avoid buying new things (e.g. clothing, luxury items)”, and “Buy or sell things on peer-to-peer websites (e.g. eBay)”. These behaviours were chosen based on their mitigation potential [52]. The measure showed good internal consistency (Chronbach’s $\:\mathrm{a}$ = 0.85). Likelihood of engaging in public-sphere behaviours was close to the midpoint (M = 2.95, SD = 0.75).

#### Climate activism (public-sphere behaviour)

An overall measure of climate activism was created from three items. Respondents were asked “Please indicate how likely or unlikely you are to take each of the following actions in the next 12 months?” (from 1 very unlikely to 5 very likely): “Support non-violent but disruptive climate protests”, “Persuade relatives or friends to reduce their carbon emissions” and “Take part in community action for environmental initiatives”. These items were chosen to represent different behaviours which share the common aim of generating support for climate action, in line with recent literature [53]. The scale showed good internal consistency (Chronbach’s *a* = 0.81). Overall likelihood of engaging in climate activism was close to the midpoint (M = 2.61, SD = 1.11).

#### Sociodemographics

The study further included the sociodemographic variables of gender, age and education as covariates (see Table 1). Gender included male and female categories; age was inlcuded as a continuous variable; and education included the categories of ‘no formal education, ‘high school or secondary school qualification’, ‘undergraduate/college degree level’ and ‘graduate/postgraduate degree level (e.g., Masters, PhD)’.

### Ethical aspects

The study was approved by Cardiff University’s School of Psychology Ethics Committee and complied with ethical guidelines. Informed consent was obtained from participants prior to data collection. All data were collected anonymously and stored securely. Given the inclusion of potentially sensitive questions (e.g., climate anxiety), participants were informed they could skip any questions they did not wish to answer and could withdraw from the study at any time, without penalty. Respondents were provided with an explanation of the study purpose at the end of the survey, and were given contact details for the research team and ethics committee, in case they required further information or had any concerns.

### Statistical analyses

Multiple regression was used to test the relationships between climate change concern, climate anxiety, and climate action (private-sphere and activism respectively). Assumptions for each regression model were tested before proceeding with analysis. First, multinomial regression was conducted to test whether climate concern predicted mild and high climate anxiety (with no climate anxiety as the reference category). Next, multiple linear regression was conducted to assess the total effect of climate concern on private-sphere behaviours and climate activism respectively, without controlling for climate anxiety. Multiple linear regression was then conducted to assess the direct effect of climate concern on private-sphere behaviours and climate activism when controlling for climate anxiety. Sobel tests were used to test for indirect effects of climate concern on climate action (private-sphere and activism respectively) through climate anxiety. Each regression model was then repeated with the inclusion of interaction terms age and climate concern, in addition to age and climate anxiety, to test for moderation effects. In each model, age, gender, education, and survey wave, were included as co-variates. Data was analysed using SPSS statistics for Mac.

## Results

### Climate concern and climate anxiety

First, multinomial regression was conducted to test whether climate concern is associated with climate anxiety. As shown in Table 2, there was a significant relationship between climate concern and climate anxiety (*p*s < 0.001), where the likelihood of having mild (OR = 2.48, 95% CI [2.18, 2.81]) and high (OR = 3.08, 95% CI [2.53, 3.74]) climate anxiety (compared to no climate anxiety) increased with climate concern. Age was also a significant predictor of mild and high climate anxiety (*p*s < 0.001), where the likelihood of having mild climate anxiety (OR = 0.98 95% CI [0.97, 0.99]) and high climate anxiety (OR = 0.94, 95% CI [0.93, 0.95]) significantly decreased as age increased.

**Table 2:** Climate concern as a predictor of climate anxiety

	B	SEB	EXP(B)	95% CI		B	SEB	EXP(B)	95% CI	
				Lower	Upper				Lower	Upper
	Mild climate anxiety^1^					High climate anxiety				
Intercept	−2.13	0.42	-	-	-	−1.51	0.64	-	-	-
Age	−0.02	0.00	0.98^***^	0.97	0.99	−0.06	0.01	0.94^***^	0.93	0.95
Education^2^ High school or secondary school qualification	0.44	0.29	1.55	0.89	2.72	−0.16	0.45	0.85	0.35	2.06
Education Undergraduate/college degree level	0.54	0.29	1.71	0.96	3.03	0.30	0.46	1.36	0.56	3.31
Education Graduate/Postgraduate degree level (e.g., Masters, PhD)	0.47	0.32	1.60	0.86	2.98	0.70	0.48	2.01	0.79	5.1
Gender^3^ Female	0.01	0.11	1.01	0.81	1.25	−0.24	0.17	0.79	0.57	1.09
Survey Wave^4^ 2023	0.01	0.11	1.01	0.82	1.25	−0.73	0.17	0.48^***^	0.35	0.67
Climate Concern	0.91	0.07	2.48^***^	2.18	2.81	1.12	0.10	3.08^***^	2.53	3.74

^***^ < *p* <.001

^1^Reference for anxiety is no anxiety

^2^Reference for education is no formal education

^3^Reference for gender is male

^4^Reference for survey wave is 2022

### Climate concern, climate anxiety and private-sphere behaviour

Next, multiple linear regression was conducted to assess the total effect of climate concern on private-sphere behaviour, without controlling for climate anxiety. The model significantly predicted likelihood of engaging in private-sphere behaviour (*F* (7, 1979) = 114.24, *p* <.001) and explained 29% of variance after accounting for the number of predictors and sample size (R^2^ = 0.29, adjusted R^2^ = 0.29). As shown in Table 3, climate concern significantly predicted increased likelihood of engaging in private-sphere behaviour (*p* <.001). Age was also a significant predictor (*p* <.001), with likelihood of performing private-sphere behaviour decreasing as age increased.

**Table 3:** Total and direct effects of climate concern on likelihood of engaging in Private-Sphere behaviours

	B	SEB	β	95% CI		B	SEB	β	95% CI	
				Lower	Upper				Lower	Upper
	Total effect of climate concern					Direct effect of climate concern				
Constant	2.15	0.09	-	1.96	2.33	1.96	0.09	-	1.78	2.13
Age	−0.01	0.00	−0.21^***^	−0.01	−0.01	−0.01	0.00	−0.12^***^	−0.01	0.00
Education^1^ High school/secondary school	0.07	0.06	0.05	−0.04	0.19	0.08	0.05	0.05	−0.03	0.18
Undergraduate/college degree	0.16	0.06	0.10^**^	0.05	0.28	0.14	0.06	0.09^*^	0.03	0.25
Graduate/Postgraduate degree	0.33	0.07	0.15^***^	0.20	0.46	0.28	0.06	0.13^***^	0.16	0.41
Gender^2^ Female	0.08	0.03	0.05^**^	0.02	0.14	0.09	0.03	0.06^**^	0.03	0.14
Survey Wave^3^ 2023	−0.08	0.03	−0.05^**^	−0.13	−0.02	−0.04	0.03	−0.03	−0.09	0.01
Climate Concern	0.34	0.02	0.43^***^	0.31	0.37	0.25	0.02	0.32^***^	0.22	0.28
Mild Climate Anxiety	-	-	-	-	-	0.35	0.03	0.23^***^	0.29	0.41
High Climate Anxiety	-	-	-	-	-	0.75	0.05	0.35^***^	0.66	0.84

^*^ < *p* <.05, ^**^ *p* <.01, ^***^ *< p* <.001

^1^Reference for education is no formal education

^2^Reference for gender is male

^3^Reference for survey wave is 2022

Linear regression was then conducted to investigate whether climate anxiety would mediate the relationship between climate concern and private-sphere behavioural likelihood. The overall model was significant (*F* (9, 1977) = 131.32, *p* <.001) and explained 37% of variance after accounting for the number of predictors and sample size (R^2^ = 0.37, adjusted R^2^ = 0.37). Climate anxiety was a significant predictor, where respondents with mild and high climate anxiety were significantly more likely to engage in private-sphere behaviour than those with no climate anxiety (*ps* < 0.001). A stronger effect size was observed for high climate anxiety (B = 0.75) compared to mild climate anxiety (B = 0.35).

**Fig. 1:**
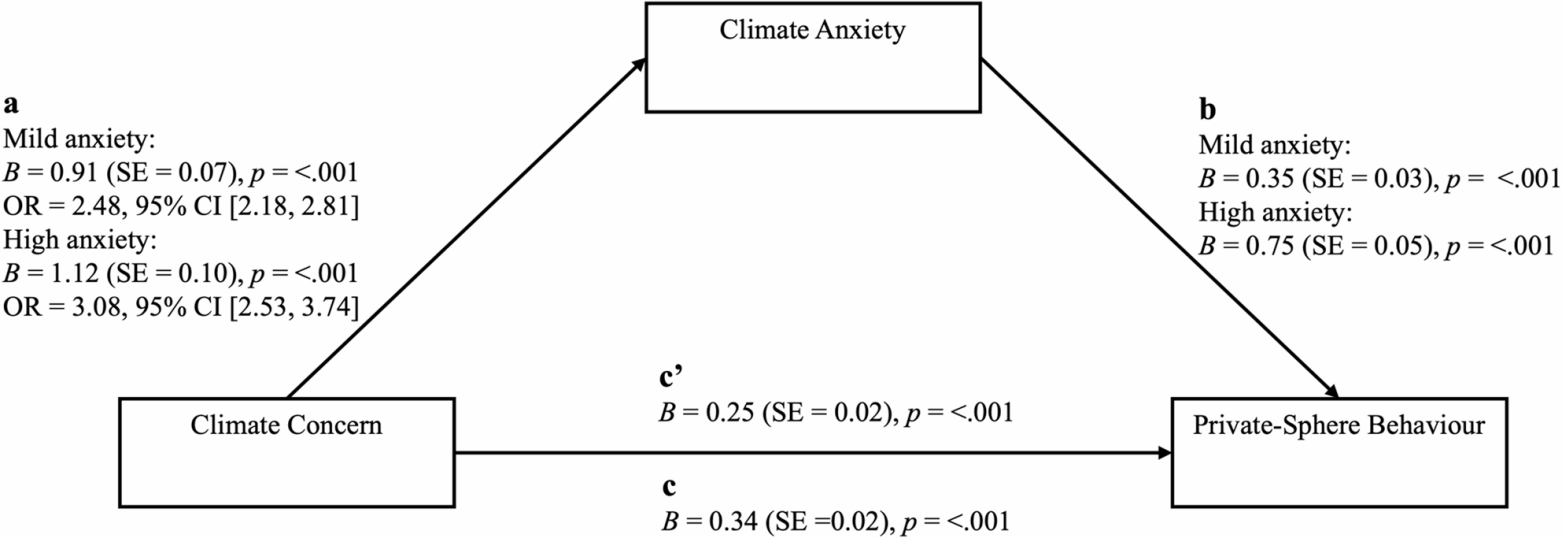
Climate anxiety as a mediator of climate concern and likelihood of engaging in private-sphere behaviour

Climate concern was still significantly associated with an increased likelihood of engaging in private-sphere behaviour when controlling for climate anxiety (*p* <.001). However, observation of the coefficients showed a decreased effect size of climate concern when observing the total effect of climate concern with climate anxiety (B = 0.25) compared to the direct effect of climate concern (B = 0.34), indicating partial mediation. The Sobel test was used to assess the indirect effect of climate concern on private-sphere behavioural likelihood through climate anxiety. The results showed a significant indirect effect of climate concern on likelihood of engaging in private-sphere behaviour through both mild climate anxiety (B = 0.31, 95% CI [0.24, 0.39], *p* <.001) and high climate anxiety (B = 0.84, 95% CI [0.66, 1.02], *p* <.001), evidencing partial mediation^1^ (see Figure. 1).

### Climate concern, climate anxiety and climate activism

Analysis was then conducted to test the relationships between climate concern, climate anxiety, and climate activism. Multiple linear regression was conducted to assess the total effect of climate concern on likelihood of engaging in climate activism. The model significantly predicted likelihood of engaging in climate activism (*F* (7, 1972) = 131.78, *p* <.001) and explained 32% of variance after accounting for the number of predictors and sample size (R^2^ = 0.32, adjusted R^2^ = 0.32). As shown in Table 4, climate concern significantly predicted increased likelihood of engaging in climate activism (*p* <.001). Age was also a significant predictor, with climate activism decreasing with age (*p* <.001).

**Table 4:** Total and direct effects of climate concern on likelihood of engaging in climate activism

	B	SEB	β	95% CI		B	SEB	β	95% CI	
				Lower	Upper				Lower	Upper
	Total effect of climate concern					Direct effect of climate concern				
Constant	1.53	0.14	-	1.26	1.80	1.21	0.13	-	0.97	1.46
Age	−0.02	0.00	−0.26^**^	−0.02	−0.01	−0.01	0.00	−0.16^***^	−0.01	−0.01
Education^1^ High school/secondary school	0.14	0.08	0.06	−0.03	0.30	0.15	0.08	0.07	0.00	0.29
Undergraduate/college degree	0.30	0.09	0.13^***^	0.14	0.47	0.27	0.08	0.11^***^	0.11	0.42
Graduate/Postgraduate degree	0.45	0.10	0.14^***^	0.26	0.64	0.37	0.09	0.11^***^	0.19	0.54
Gender^2^ Female	0.04	0.04	0.02	−0.04	0.12	0.05	0.04	0.02	−0.02	0.13
Survey Wave^3^ 2023	−0.06	0.04	−0.03	−0.14	0.03	0.01	0.04	0.00	−0.07	0.08
Climate Concern	0.51	0.02	0.44^***^	0.47	0.56	0.36	0.02	0.31^***^	0.32	0.41
Mild Climate Anxiety	-	-	-	-	-	0.63	0.04	0.29^***^	0.55	0.72
High Climate Anxiety	-	-	-	-	-	1.23	0.07	0.39^***^	1.10	1.36

^***^ *< p* <.001

^1^Reference for education is no formal education

^2^Reference for gender is male

^3^Reference for survey wave is 2022

Linear regression was conducted to investigate whether climate anxiety predicts climate activism and climate anxiety mediates the relationship between climate concern and climate activism. The overall model significantly predicted likelihood of engaging in climate activism (*F* (9, 1970) = 165.80, *p* <.001) and explained 43% of variance after accounting for the number of predictors and sample size (R^2^ = 0.43, adjusted R^2^ = 0.43). As shown in Table 4, climate anxiety was a significant predictor, where respondents with mild and high climate anxiety were significantly more likely to engage in climate activism compared to those with no climate anxiety (*ps* < 0.001). A stronger effect size was also observed for those with high climate anxiety (B = 1.23) compared to mild climate anxiety (B = 0.63). As with the previous models, likelihood of engaging in climate activism significantly decreased with age (*p* <.001).

**Fig. 2:**
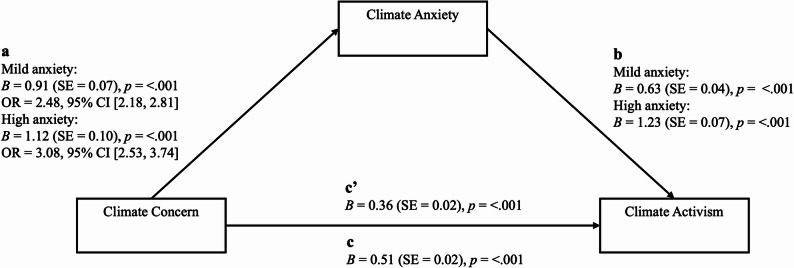
Climate anxiety as a mediator of climate concern and likelihood of engaging in climate activism

Climate concern was still significantly associated with an increased likelihood of engaging in climate activism after controlling for climate anxiety (*p* <.001). However, the coefficients show a decreased effect size of climate concern when observing the direct effect of climate concern with climate anxiety (B = 0.36) compared to the total effect of climate concern (B = 0.51), indicating partial mediation. The Sobel test showed a significant indirect effect of climate concern on likelihood of engaging in climate activism through mild climate anxiety (B = 0.57, 95% CI [0.46, 0.69], *p* <.001) and high climate anxiety (B = 1.38, 95% CI [1.10, 1.66], *p* <.001), evidencing partial mediation (see Figure. 2).

Finally, analyses were conducted to investigate whether age moderates the strength of the above relationships. Age and climate concern were mean centred and used to compute interaction terms. Each of the above regression models were repeated with the respective interaction term included (see Tables 5, 6 and 7). The interaction between age and climate concern was not a significant predictor of mild or high climate anxiety (*ps* > 0.05). The interaction between age and climate anxiety was also not a significant predictor of likelihood of engaging in private-sphere behaviour or climate activism (*ps* > 0.05). Observation of simple slopes further showed no indication of interaction effects. Therefore, there was no evidence that age impacted upon the relationships between climate concern, climate anxiety and likelihood of engaging in pro-environmental behaviours (private-sphere behaviour and climate activism respectively).

**Table 5:** Interaction terms from regression models testing for moderation of age on climate anxiety

Predictor	Outcome	*B*	*SEB*	EXP(B)	95% CI	
					Lower	Upper
Climate Concern*Age^1^	Mild climate anxiety^1^	0.00	0.00	1.00	0.99	1.01
	High climate anxiety	0.00	0.01	1.00	0.99	1.01

The full regression model can be found in Appendix A

^1^ Mean centred

^2^The reference category for climate anxiety is no anxiety

**Table 6:** Testing for moderation of age on likelihood of engaging in Private-Sphere behaviours

Predictor	B	SEB	β	95% CI	
				Lower	Upper
Climate concern * Age^1^	0.00	0.00	0.00	0.00	0.00
Mild climate anxiety* Age^1^	0.00	0.00	−0.02	−0.01	0.00
High climate anxiety * Age^1^	0.00	0.00	−0.03	−0.01	0.00

The full regression model can be found in Appendix B

^1^ Mean centred

**Table 7:** Interaction terms from regression models testing for moderation of age on likelihood of engaging in climate activism

Predictor	B	SEB	β	95% CI	
				Lower	Upper
Climate concern * Age^1^	0.00	0.00	0.01	0.00	0.00
Mild climate anxiety * Age^1^	−0.01	0.00	−0.05	−0.01	0.00
High climate anxiety * Age^1^	0.00	0.00	−0.02	−0.01	0.01

The full regression model can be found in Appendix C

^1^ Mean centred

## Discussion

The present study adds to emerging literature on climate anxiety by examining the relationship between climate anxiety and different types of pro-environmental behaviour, while differentiating its effects from that of climate concern, and assessing potential moderating effects of age. The results show that individuals concerned about climate change were significantly more likely to experience climate anxiety. Both climate concern and climate anxiety were in turn associated with an increased likelihood of engaging in private-sphere behaviours and climate activism, with the effects of climate concern being driven in part by climate anxiety. Younger participants were significantly more likely to experience climate anxiety than older respondents, supporting emerging literature demonstrating the toll of climate change on young people [7, 9, 10]. However, the mechanisms of climate change concern and anxiety on motivating an increased likelihood of engaging in climate action were consistent across respondents of different ages. Therefore, the lower likelihood of engaging in private-sphere behaviours and climate activism as age increased identified in this study may be attributed to other factors. The results are discussed in detail below.

The results show that being concerned about climate change is sufficient to motivate an increased likelihood of engaging in private-sphere and climate activist behaviours. This suggests that the effects of concern for climate change may not be limited to low-cost and low-effort behaviours as previously reported [29, 30]. The finding that climate concern was associated with climate anxiety supports recent literature demonstrating a hierarchical relationship between cognitive and affective components of climate change engagement (see[47]). Moreover, the effects of climate concern were in part driven by climate anxiety, demonstrating that stronger affective responses to climate change have a greater impact on driving pro-environmental behaviour. Overall levels of climate concern were higher than overall levels of climate anxiety, supporting recent literature also demonstrating lower levels of climate anxiety than climate concern in the UK [9]. This indicates that levels of general concern or worry about climate change may be more common than more intense forms of climate anxiety (i.e. resulting in impaired functioning).

The results showed that 63% of respondents were experiencing climate anxiety to some extent, with the majority experiencing a mild level and a small proportion experiencing a high level of climate anxiety. This indicates that while climate anxiety may not affect individuals consistently, it is still pervasive and being felt to some degree among the UK population. Climate anxiety was found to increase the likelihood of engaging in private-sphere behaviour as well as climate activism consistently among those experiencing mild and high levels of climate anxiety, compared to those experiencing no anxiety. This shows that even very mild climate anxiety can be transformed into meaningful action, with the potential to mitigate rising emissions through encouraging change at an individual, community, and societal level [49]. However, a stronger effect size was shown for high levels of climate anxiety compared to mild levels of anxiety, indicating that higher levels of anxiety have an even stronger effect on motivating pro-environmental behaviours. This finding has implications for measurement of climate anxiety, as continuous measures may not fully capture how varying degrees of climate anxiety may affect individuals and their subsequent behavioural engagement in different ways.

The present results lend support to arguments that climate anxiety can have an adaptive function, even if climate anxiety is conceptualised through impaired functioning. In line with basic emotion theory, climate anxiety was found to increase relevant climate mitigation behaviours, which may in turn help to reduce one’s anxiety. For example, engaging in collective climate action can have positive impacts on wellbeing and mental health, by helping concerned individuals transform their distress into something positive [46]. This is supported by evidence that engaging in collective action can reduce the association between climate change anxiety and depression symptoms, with no such effect being shown for individual action [8]. Collective action has also been associated with lower levels of climate anxiety [54]. Therefore, the finding that climate concern and anxiety led to an increased likelihood of engaging in climate activism in the present study is a positive one, as affective responses to climate change can be transformed into meaningful action, potentially then acting as a ‘buffer’ against climate-related distress [8]. With the above findings, this supports arguments that strategies to promote psychological wellbeing and active engagement should focus on cultivating the capacity to learn from worry and channel it into constructive actions, ensuring it does not lead to unhealthy coping mechanisms [43, 55].

It is important to note that the behaviours used in this study tended to involve relatively high effort actions, such as following a vegan diet, living car free, and taking part in community action. However, climate anxiety may have different effects on low effort and high effort behaviours. For example, Clayton and Karazsia [7] found no association between climate anxiety and relatively low effort behaviours such as recycling and turning off lights, while Whitmarsh et al. [9] found that climate anxiety predicted high effort but not low effort actions. It is possible that individuals experiencing climate anxiety may be more willing to perform high effort behaviours as a means of managing their anxiety, as high effort behaviours (e.g. living car free) tend to be more impactful in terms of reducing greenhouse gas emissions than low effort behaviours (e.g. recycling; [56]). Moreover, behaviours differing in terms of impact may also have differing impacts on subsequent wellbeing. For example, A meta-analysis of 71 studies involving 391,379 participants found that low-impact pro-environmental behaviours (e.g., recycling or using reusable bags) were significantly positively associated with subjective well-being, while no such association was found for high-impact behaviours (e.g., adopting a vegetarian diet or commuting via public transport; [57]). Future research could further investigate the effects of climate anxiety on private- and public-sphere behaviours differing in terms of difficulty to perform and impact on emission reductions. This would provide further insight into the capacity of climate anxiety to motivate climate action, subsequent mitigation potential, and impacts on wellbeing.

The impact of climate change on the mental health of young people is currently poorly understood, though emerging evidence shows climate anxiety may disproportionally affect younger age groups [58]. This is supported in the present study, which demonstrated higher levels of climate anxiety among younger respondents. The likelihood of engaging in climate action also significantly decreased as age increased in our sample. Yet, the effects of climate change concern and anxiety on motivating private-sphere behaviours and climate activism were consistent across respondents of different ages. The decreased likelihood of engaging in climate relevant behaviours among older respondents may therefore be attributed to other factors. For example, past research has shown generational differences in climate-related beliefs, including increased scepticism and lower risk perceptions, in addition to lower levels of climate-related emotions, such as fear and outrage, among older generations compared to younger generations [47, 50]. Understanding the mechanisms underlying generational differences in climate change engagement would be a worthwhile avenue of future research, to determine ways of motivating greater engagement across generations, subsequently reducing the burden on young people.

This study is not without limitations. While this study investigated different types of pro-environmental behaviour, a drawback is measuring the likelihood of engaging in different actions, which may not accurately represent real behavioural engagement. This could be addressed in future studies by asking respondents to report their behaviour retrospectively (e.g., over the last 12 months) or by measuring behaviour directly using observational methods (e.g., surveying participants of climate protests). Although the sample was broadly representative of the UK adult population, certain demographic groups were slightly over- or underrepresented. Specifically, older adults were overrepresented, while respondents of Asian and Black backgrounds were slightly underrepresented, compared to UK census estimates [59, 60]. Therefore, while the findings are likely to generalise well to the wider population, caution is warranted when interpreting results for these specific subgroups. Finally, this study involved the development of new scales for private-sphere behaviour and climate activism, which are not explicitly validated. Scales were developed based on similar past research and showed good internal reliability. However, it is important to note that the development of these scales required subjective decisions regarding item inclusion. These decisions are inevitably informed by our disciplinary backgrounds, and interpretations of the existing literature [61].

## Conclusion

Little is currently known about how climate change concern and climate anxiety may interact with each other to motivate or hinder environmental action. This paper addresses this gap and contributes to existing literature by disentangling the effects of climate change concern and climate anxiety on different types of pro-environmental behaviour, using UK survey data. The results showed that individuals concerned about climate change were significantly more likely to experience climate anxiety and were subsequently more likely to engage in climate action. The effects of climate change concern and anxiety on climate action were consistent across respondents of different ages. Climate anxiety therefore may be adaptive in encouraging relevant climate action.

## Supplementary Information

Supplementary Material 1

Supplementary Material 2

## Data Availability

The dataset generated and analysed during the current study will be available to the UK Data Service (https://ukdataservice.ac.uk) as per the requirement for ESRC-funded projects. The data will be deposited after publication. In case of delay between publication and depositing the data, data files can be requested from the authors of the paper.

## Footnotes

1 Private-sphere behaviours were grouped into categories relating to diet, transport, heating, and material consumption, to identify whether the results would differ according to the type of behaviour performed. The results replicated the above findings demonstrating a consistent indirect effect of climate concern on private-sphere behavioural likelihood through climate anxiety, with the exception of heating which was derived from a single item and therefore may lack reliability (see supplementary material).

## References

[1] Calvin K, Dasgupta D, Krinner G, Mukherji A, Thorne PW, Trisos C et al. IPCC, 2023: Climate Change 2023: Synthesis Report. Contribution of Working Groups I, II and III to the Sixth Assessment Report of the Intergovernmental Panel on Climate Change [Core Writing Team, H. Lee and J. Romero, editors]. IPCC, Geneva, Switzerland. First. Intergovernmental Panel on Climate Change (IPCC); 2023 Jul [cited 2024 Dec 17]. Available from: https://www.ipcc.ch/report/ar6/syr/

[2] Morganstein JC, Ursano RJ. Ecological disasters and mental health: Causes, Consequences, and interventions. Front Psychiatry. 2020;11:1. 32116830 10.3389/fpsyt.2020.00001 PMC7026686

[3] Met Office. [cited 2024 Dec 17]. 2023 was second warmest year on record for UK. Available from: https://www.metoffice.gov.uk/about-us/news-and-media/media-centre/weather-and-climate-news/2023/2023-was-second-warmest-year-on-record-for-uk

[4] Wolstenholme E, Steentjes K, Demski C, Poortinga W. Public perceptions of climate change and policy action in the UK, China, Sweden and Brazil from 2020–2023. Report No.: 26. Available from: https://cast.ac.uk/wp-content/uploads/2024/04/CAST-the-centre-for-climate-change-and-social-transformations-cast-briefing-26-public-perceptions-of-climate-change-and-policy-action-in-the-uk-china-sweden-and-brazil-from-2020-to-2023.pdf

[5] Clayton S. Climate anxiety: psychological responses to climate change. J Anxiety Disord. 2020;74:102263. 32623280 10.1016/j.janxdis.2020.102263

[6] Tam KP, Chan HW, Clayton S. Climate change anxiety in China, India, Japan, and the united States. J Environ Psychol. 2023;87:101991.

[7] Clayton S, Karazsia BT. Development and validation of a measure of climate change anxiety. J Environ Psychol. 2020;69:101434.

[8] Schwartz SEO, Benoit L, Clayton S, Parnes MF, Swenson L, Lowe SR. Climate change anxiety and mental health: environmental activism as buffer. Curr Psychol. 2023;42(20):16708–21. 10.1007/s12144-022-02735-6 PMC8883014 35250241

[9] Whitmarsh L, Player L, Jiongco A, James M, Williams M, Marks E, et al. Climate anxiety: what predicts it and how is it related to climate action? J Environ Psychol. 2022;83:101866.

[10] Hickman C, Marks E, Pihkala P, Clayton S, Lewandowski RE, Mayall EE, et al. Climate anxiety in children and young people and their beliefs about government responses to climate change: a global survey. Lancet Planet Health. 2021;5(12):e863–73. 34895496 10.1016/S2542-5196(21)00278-3

[11] Verplanken B, Marks E, Dobromir AI. On the nature of eco-anxiety: how constructive or unconstructive is habitual worry about global warming? J Environ Psychol. 2020;72:101528.

[12] Ojala M, Cunsolo A, Ogunbode CA, Middleton J. Anxiety, Worry, and grief in a time of environmental and climate crisis: A narrative review. Annu Rev Environ Resour. 2021;46(1):35–58.

[13] Pihkala P. Toward a taxonomy of climate emotions. Front Clim. 2022;3:738154.

[14] Clayton S, Manning CM, Speiser M, Hill AN. Mental Health and Our Changing Climate: Impacts, Inequities, Responses. Washington, D.C: American Psychological Association, and ecoAmerica.; 2021. Available from: https://www.apa.org/news/press/releases/mental-health-climate-change.pdf

[15] Cunsolo A, Ellis NR. Ecological grief as a mental health response to climate change-related loss. Nat Clim Change. 2018;8(4):275–81.

[16] Albrecht G, Sartore GM, Connor L, Higginbotham N, Freeman S, Kelly B, et al. Solastalgia: the distress caused by environmental change. Australas Psychiatry. 2007;15(1suppl):S95–8. 18027145 10.1080/10398560701701288

[17] Pihkala P. Anxiety and the ecological crisis: an analysis of Eco-Anxiety and climate anxiety. Sustainability. 2020;12(19):7836.

[18] Keltner D, Sauter D, Tracy J, Cowen A. Emotional expression: advances in basic emotion theory. J Nonverbal Behav. 2019;43(2):133–60. 31395997 10.1007/s10919-019-00293-3 PMC6687086

[19] Steimer T. The biology of fear- and anxiety-related behaviors. Dialogues Clin Neurosci. 2002;4(3):231–49. 22033741 10.31887/DCNS.2002.4.3/tsteimer PMC3181681

[20] Barlow DH, Durand VM, Hofmann SG. Abnormal Psychology: An Integrative Approach by David H. Barlow. Abnorm Psychol Integr Approach. 2019 [cited 2024 Dec 17];8. Available from: https://www.academia.edu/43163654/Abnormal_Psychology_An_Integrative_Approach_by_David_H_Barlow

[21] Barlow DH. Anxiety and its disorders: the nature and treatment of anxiety and panic. Guilford Press; 2004. p. 724.

[22] Crandon TJ, Scott JG, Charlson FJ, Thomas HJ. A theoretical model of climate anxiety and coping. Discov Psychol. 2024;4(1):94.

[23] Bouman T, Verschoor M, Albers CJ, Böhm G, Fisher SD, Poortinga W, et al. When worry about climate change leads to climate action: how values, worry and personal responsibility relate to various climate actions. Glob Environ Change. 2020;62:102061.

[24] Coelho F, Pereira MC, Cruz L, Simões P, Barata E. Affect and the adoption of pro-environmental behaviour: A structural model. J Environ Psychol. 2017;54:127–38.

[25] Semenza JC, Hall DE, Wilson DJ, Bontempo BD, Sailor DJ, George LA. Public perception of climate change. Am J Prev Med. 2008;35(5):479–87. 18929974 10.1016/j.amepre.2008.08.020

[26] Sundblad EL, Biel A, Gärling T. Intention to change activities that reduce carbon dioxide emissions related to worry about global climate change consequences. Eur Rev Appl Psychol. 2014;64(1):13–7.

[27] Bamberg S. How does environmental concern influence specific environmentally related behaviors? A new answer to an old question. J Environ Psychol. 2003;23(1):21–32.

[28] Jakučionytė-Skodienė M, Liobikienė G. The changes in climate change Concern, responsibility assumption and impact on climate-friendly behaviour in EU from the Paris agreement until 2019. Environ Manage. 2022;69(1):1–16. 34993591 10.1007/s00267-021-01574-8 PMC8739017

[29] Tobler C, Visschers VHM, Siegrist M. Addressing climate change: determinants of consumers’ willingness to act and to support policy measures. J Environ Psychol. 2012;32(3):197–207.

[30] Whitmarsh L. Behavioural responses to climate change: asymmetry of intentions and impacts. J Environ Psychol. 2009;29(1):13–23.

[31] Ogunbode CA, Doran R, Hanss D, Ojala M, Salmela-Aro K, Van Den Broek KL, et al. Climate anxiety, wellbeing and pro-environmental action: correlates of negative emotional responses to climate change in 32 countries. J Environ Psychol. 2022;84:101887.

[32] Sangervo J, Jylhä KM, Pihkala P. Climate anxiety: conceptual considerations, and connections with climate hope and action. Glob Environ Change. 2022;76:102569.

[33] Soutar C, Wand APF. Understanding the spectrum of anxiety responses to climate change: A systematic review of the qualitative literature. Int J Environ Res Public Health. 2022;19(2):990. 35055813 10.3390/ijerph19020990 PMC8776219

[34] Pihkala P. The process of Eco-Anxiety and ecological grief: A narrative review and a new proposal. Sustainability. 2022;14(24):16628.

[35] Stanley SK, Hogg TL, Leviston Z, Walker I. From anger to action: differential impacts of eco-anxiety, eco-depression, and eco-anger on climate action and wellbeing. J Clim Change Health. 2021;1:100003.

[36] Innocenti M, Santarelli G, Lombardi GS, Ciabini L, Zjalic D, Di Russo M, et al. How can climate change anxiety induce both Pro-Environmental behaviours and Eco-Paralysis? The mediating role of general Self-Efficacy. Int J Environ Res Public Health. 2023;20(4):3085. 36833780 10.3390/ijerph20043085 PMC9960236

[37] Albrecht G. Chronic environmental change: emerging ‘psychoterratic’ syndromes. Climate change and human well-being: global challenges and opportunities. New York, NY, US: Springer Science + Business Media; 2011. pp. 43–56. (International and cultural psychology).

[38] Nielsen KS, Cologna V, Bauer JM, Berger S, Brick C, Dietz T, et al. Realizing the full potential of behavioural science for climate change mitigation. Nat Clim Change. 2024;14(4):322–30.

[39] Stern PC. New environmental theories: toward a coherent theory of environmentally significant behavior. J Soc Issues. 2000;56(3):407–24.

[40] Balzekiene A, Telesiene A. Explaining private and public sphere personal environmental behaviour. Soc Sci. 2012;74(4):7–19.

[41] Bamberg S, Rees JH, Schulte M. Environmental protection through societal change. In: Psychology and Climate Change. Elsevier; 2018 [cited 2024 Dec 17]. pp. 185–213. Available from: https://linkinghub.elsevier.com/retrieve/pii/B9780128131305000084

[42] Whitmarsh L, Poortinga W, Capstick S. Behaviour change to address climate change. Curr Opin Psychol. 2021;42:76–81. 33991862 10.1016/j.copsyc.2021.04.002

[43] Latkin C, Dayton L, Scherkoske M, Countess K, Thrul J. What predicts climate change activism? An examination of how depressive symptoms, climate change distress, and social norms are associated with climate change activism. J Clim Change Health. 2022;8:100146. 10.1016/j.joclim.2022.100146 PMC9910281 36777085

[44] Skapinakis P. Spielberger State-Trait Anxiety Inventory. In: Michalos AC, editor. Encyclopedia of Quality of Life and Well-Being Research. Dordrecht: Springer Netherlands; 2014 [cited 2024 Dec 17]. pp. 6261–4. Available from: 10.1007/978-94-007-0753-5_2825

[45] Kleres J, Wettergren Å. Fear, hope, anger, and guilt in climate activism. Soc Mov Stud. 2017;16(5):507–19.

[46] Budziszewska M, Jonsson SE. From climate anxiety to climate action: an existential perspective on climate change concerns within psychotherapy. J Humanist Psychol , *0**(**0**)*. 2021;0022167821993243.

[47] Poortinga W, Demski C, Steentjes K. Generational differences in climate-related beliefs, risk perceptions and emotions in the UK. Commun Earth Environ. 2023;4(1):229.

[48] Zhou S, Yu B, Zhang Y. Global concurrent climate extremes exacerbated by anthropogenic climate change. Sci Adv. 2023;9(10):eabo1638. 36897946 10.1126/sciadv.abo1638 PMC10005174

[49] Milfont TL. The interplay between Knowledge, perceived Efficacy, and concern about global warming and climate change: A One-Year longitudinal study. Risk Anal. 2012;32(6):1003–20. 22489642 10.1111/j.1539-6924.2012.01800.x

[50] Poortinga W, Whitmarsh L, Steg L, Böhm G, Fisher S. Climate change perceptions and their individual-level determinants: A cross-European analysis. Glob Environ Change. 2019;55:25–35.

[51] Swim JK, Aviste R, Lengieza ML, Fasano CJ. OK boomer: A decade of generational differences in feelings about climate change. Glob Environ Change. 2022;73:102479.

[52] Ivanova D, Barrett J, Wiedenhofer D, Macura B, Callaghan M, Creutzig F. Quantifying the potential for climate change mitigation of consumption options. Environ Res Lett. 2020;15(9):093001.

[53] Kallergi A, Landeweerd L, Science, Activism, Action C. Navigating Credibility, Responsibility, and Engagement. J Acad Ethics. 2025 Apr 22 [cited 2025 Jul 8]; Available from: 10.1007/s10805-025-09626-y

[54] Lukacs JN, Bratu A, Adams S, Logie C, Tok N, McCunn LJ, et al. The concerned steward effect: exploring the relationship between climate anxiety, psychological distress, and self-reported climate related behavioural engagement. J Environ Psychol. 2023;90:102091.

[55] Ojala M. Confronting macrosocial worries: worry about environmental problems and proactive coping among a group of young volunteers. Futures. 2007;39(6):729–45.

[56] Hampton S, Whitmarsh L. Choices for climate action: A review of the multiple roles individuals play. One Earth. 2023;6(9):1157–72.

[57] Krumm L. The relationship between pro-environmental behavior, subjective well-being, and environmental impact: a meta-analysis. Environ Res Lett. 2024;19(9):094056.

[58] Ma T, Moore J, Cleary A. Climate change impacts on the mental health and wellbeing of young people: A scoping review of risk and protective factors. Soc Sci Med. 2022;301:114888. 35367905 10.1016/j.socscimed.2022.114888

[59] Population estimates for the UK, England. Wales, Scotland, and Northern Ireland - Office for National Statistics. [cited 2025 Jul 8]. Available from: https://www.ons.gov.uk/peoplepopulationandcommunity/populationandmigration/populationestimates/bulletins/annualmidyearpopulationestimates/mid2022

[60] Ethnic group, England and Wales. - Office for National Statistics. [cited 2025 Jul 8]. Available from: https://www.ons.gov.uk/peoplepopulationandcommunity/culturalidentity/ethnicity/bulletins/ethnicgroupenglandandwales/census2021

[61] Jamieson MK, Govaart GH, Pownall M. Reflexivity in quantitative research: A rationale and beginner’s guide. Soc Personal Psychol Compass. 2023;17(4):e12735.

