# Supplementary material for: Examining the relationship between climate concern, climate anxiety and climate action in the UK
Source: BMC Psychol. 2026 Mar 3;14:731. doi: 10.1186/s40359-026-04170-9 (PMC13185269; doi:10.1186/s40359-026-04170-9)
Supplement: Supplementary file 1 — Supplementary Material 1 [file 40359_2026_4170_MOESM1_ESM.docx]

**Appendices**

**Appendix A**

*Full Regression Model with Interaction Terms Testing for Moderation of Age on Climate Anxiety*

|  | *B* | *SEB* | EXP(B) | 95% CI | | *B* | *SEB* | EXP(B) | 95% CI | |
| --- | --- | --- | --- | --- | --- | --- | --- | --- | --- | --- |
|  |  |  |  | Lower | Upper |  |  |  | Lower | Upper |
|  | Mild climate anxiety^1^ | | | | | High climate anxiety | | | | |
| Intercept | -0.07 | 0.29 | - | - | - | -0.90 | 0.45 | - | - | - |
| Education^2^ |  |  |  |  |  |  |  |  |  |  |
| High school or secondary school qualification | 0.43 | 0.29 | 1.54 | 0.88 | 2.70 | -0.17 | 0.45 | 0.85 | 0.35 | 2.06 |
| Undergraduate/college degree level | 0.53 | 0.29 | 1.69 | 0.95 | 3.00 | 0.30 | 0.46 | 1.35 | 0.55 | 3.30 |
| Graduate/Postgraduate degree level (e.g., Masters, PhD) | 0.46 | 0.32 | 1.58 | 0.85 | 2.95 | 0.69 | 0.48 | 2.00 | 0.79 | 5.09 |
| Survey Wave^3^ |  |  |  |  |  |  |  |  |  |  |
| 2023 | 0.01 | 0.11 | 1.01 | 0.82 | 1.25 | -0.73 | 0.17 | 0.48^***^ | 0.35 | 0.67 |
| Gender^4^ |  |  |  |  |  |  |  |  |  |  |
| Female | 0.01 | 0.11 | 1.01 | 0.81 | 1.25 | -0.24 | 0.17 | 0.79 | 0.57 | 1.09 |
| Climate concern^5^ | 0.91 | 0.07 | 2.49^***^ | 2.19 | 2.84 | 1.13 | 0.11 | 3.11^***^ | 2.50 | 3.86 |
| Age^5^ | -0.02 | 0.00 | 0.98^***^ | 0.97 | 0.99 | -0.06 | 0.01 | 0.94^***^ | 0.93 | 0.95 |
| Climate concern * Age^5^ | 0.00 | 0.00 | 1.00 | 0.99 | 1.01 | 0.00 | 0.01 | 1.00 | 0.99 | 1.01 |

^1^Reference for anxiety is no anxiety, ^2^Reference for education is no formal education, ^3^Reference for survey wave is 2022, ^4^Reference for gender is male,^5^Age and climate concern are mean centred, ^***^ *p* <.001, ^**^ *p* <.01, ^*^ *p< .05.*

**Appendix B**

*Full Regression Model with Interaction Terms Testing for Moderation of Age on Likelihood of Engaging in Private-Sphere Behaviours*

|  | *B* | *SEB* | *β* | 95% CI | |
| --- | --- | --- | --- | --- | --- |
|  |  |  |  | Lower | Upper |
| Constant | 2.53 | 0.06 | - | 2.42 | 2.64 |
| Education^1^ |  |  |  |  |  |
| High school or secondary school qualification | 0.08 | 0.05 | 0.05 | -0.03 | 0.18 |
| Education  Undergraduate/college degree level | 0.14 | 0.06 | 0.09^*^ | 0.03 | 0.25 |
| Education  Graduate/Postgraduate degree level (e.g., Masters, PhD) | 0.28 | 0.06 | 0.13^***^ | 0.16 | 0.40 |
| Gender^2^ |  |  |  |  |  |
| Female | 0.09 | 0.03 | 0.06^**^ | 0.03 | 0.14 |
| Survey Wave^3^ |  |  |  |  |  |
| 2023 | -0.04 | 0.03 | -0.03 | -0.09 | 0.02 |
| Climate anxiety^4^ |  |  |  |  |  |
| Mild climate anxiety | 0.35 | 0.03 | 0.23^***^ | 0.29 | 0.41 |
| High climate anxiety | 0.73 | 0.05 | 0.34^***^ | 0.62 | 0.83 |
| Age^5^ | 0.00 | 0.00 | -0.10^**^ | -0.01 | 0.00 |
| Climate concern^5^ | 0.25 | 0.02 | 0.32^***^ | 0.22 | 0.28 |
| Age*Mild climate anxiety^5^ | 0.00 | 0.00 | -0.02 | -0.01 | 0.00 |
| Age*High climate anxiety^5^ | 0.00 | 0.00 | -0.03 | -0.01 | 0.00 |
| Age*Climate concern^5^ | 0.00 | 0.00 | 0.00 | 0.00 | 0.00 |

^1^ Reference for education is no formal education, ^2^Reference for gender is male, ^3^Reference for survey wave is 2022, ^4^Reference for anxiety is no anxiety, ^5^Age and climate concern are mean centred, ^***^ *p* <.001, ^**^ *p* <.01, ^*^ *p < .05 ^***^ p <.001.*

**Appendix C**

*Full Regression Model with Interaction Terms Testing for Moderation of Age on Likelihood of Engaging in Climate Activism*

|  | *B* | *SEB* | *β* | 95% CI | |
| --- | --- | --- | --- | --- | --- |
|  |  |  |  | Lower | Upper |
| Constant | 1.88 | 0.08 | - | 1.72 | 2.03 |
| Education^1^ |  |  |  |  |  |
| High school or secondary school qualification | 0.15 | 0.08 | 0.07^*^ | 0.00 | 0.30 |
| Education  Undergraduate/college degree level | 0.27 | 0.08 | 0.11^***^ | 0.12 | 0.42 |
| Education  Graduate/Postgraduate degree level (e.g., Masters, PhD) | 0.37 | 0.09 | 0.11^***^ | 0.20 | 0.54 |
| Gender^2^ |  |  |  |  |  |
| Female | 0.05 | 0.04 | 0.02 | -0.03 | 0.13 |
| Survey Wave^3^ |  |  |  |  |  |
| 2023 | 0.01 | 0.04 | 0.00 | -0.07 | 0.08 |
| Climate Anxiety^4^ |  |  |  |  |  |
| Mild climate anxiety | 0.65 | 0.05 | 0.29^***^ | 0.56 | 0.73 |
| High climate anxiety | 1.25 | 0.08 | 0.39^***^ | 1.10 | 1.40 |
| Age^5^ | -0.01 | 0.00 | -0.12^***^ | -0.01 | 0.00 |
| Climate concern^5^ | 0.36 | 0.02 | 0.31^***^ | 0.32 | 0.40 |
| Age*Mild climate anxiety^5^ | -0.01 | 0.00 | -0.05 | -0.01 | 0.00 |
| Age*High climate anxiety^5^ | 0.00 | 0.00 | -0.02 | -0.01 | 0.01 |
| Age*Climate concern^5^ | 0.00 | 0.00 | 0.01 | 0.00 | 0.00 |

^1^ Reference for education is no formal education, ^2^Reference for gender is male, ^3^Reference for survey wave is 2022, ^4^Reference for anxiety is no anxiety, ^5^Age and climate concern are mean centred, ^***^ *p* <.001, ^**^ *p* <.01, ^*^ *p < .05 ^***^ p <.001.*
