# Supplementary material for: Examining the relationship between climate concern, climate anxiety and climate action in the UK
Source: BMC Psychol. 2026 Mar 3;14:731. doi: 10.1186/s40359-026-04170-9 (PMC13185269; doi:10.1186/s40359-026-04170-9)
Supplement: Supplementary file 2 — Supplementary Material 2 [file 40359_2026_4170_MOESM2_ESM.docx]

**Supplementary material**

**Table S1**

Total and Direct effects of Climate Concern on Likelihood of Engaging in Private-Sphere Behaviours: Diet

|  | *B* | *SEB* | *β* | 95% CI | | *B* | *SEB* | *β* | 95% CI | |
| --- | --- | --- | --- | --- | --- | --- | --- | --- | --- | --- |
|  |  |  |  | Lower | Upper |  |  |  | Lower | Upper |
|  | Total effect of climate concern | | | | | Direct effect of climate concern | | | | |
| Constant | 1.98 | 0.10 | - | 1.78 | 2.18 | 1.80 | 0.10 | - | 1.61 | 1.99 |
| Age | 0.00 | 0.00 | -0.09^***^ | -0.01 | 0.00 | 0.00 | 0.00 | -0.01 | 0.00 | 0.00 |
| Education^1^  High school or secondary school qualification | 0.07 | 0.06 | 0.05 | -0.05 | 0.20 | 0.08 | 0.06 | 0.05 | -0.04 | 0.19 |
| Education  Undergraduate/college degree level | 0.16 | 0.07 | 0.10^*^ | 0.04 | 0.29 | 0.14 | 0.06 | 0.08^*^ | 0.02 | 0.26 |
| Education  Graduate/Postgraduate degree level (e.g., Masters, PhD) | 0.30 | 0.07 | 0.13^***^ | 0.15 | 0.44 | 0.25 | 0.07 | 0.11^***^ | 0.11 | 0.39 |
| Gender ^2^  Female | 0.17 | 0.03 | 0.11^***^ | 0.11 | 0.24 | 0.18 | 0.03 | 0.11^***^ | 0.12 | 0.24 |
| Survey Wave^3^  2023 | -0.04 | 0.03 | -0.02 | -0.10 | 0.02 | 0.00 | 0.03 | 0.00 | -0.06 | 0.06 |
| Climate Concern | 0.35 | 0.02 | 0.41^***^ | 0.31 | 0.38 | 0.27 | 0.02 | 0.32^***^ | 0.23 | 0.30 |
| Mild climate anxiety | - | - | - |  |  | 0.30 | 0.04 | 0.19^***^ | 0.23 | 0.37 |
| High climate anxiety | - | - | - |  |  | 0.71 | 0.05 | 0.31^***^ | 0.60 | 0.81 |

^1^Reference for education is no formal education, ^2^Reference for gender is male, ^3^Reference for survey wave is 2022, ^*^ *p* <.05, ^**^ *p* <.01, ^***^ *p* <.001

**Table S2**

Total and Direct effects of Climate Concern on Likelihood of Engaging in Private-Sphere Behaviours: Travel

|  | *B* | *SEB* | *β* | 95% CI | | *B* | *SEB* | *β* | 95% CI | |
| --- | --- | --- | --- | --- | --- | --- | --- | --- | --- | --- |
|  |  |  |  | Lower | Upper |  |  |  | Lower | Upper |
|  | Total effect of climate concern | | | | | Direct effect of climate concern | | | | |
| Constant | 2.21 | 0.14 | - | 1.95 | 2.48 | 1.95 | 0.13 | - | 1.70 | 2.20 |
| Age | -0.02 | 0.00 | -0.25^***^ | -0.02 | -0.01 | -0.01 | 0.00 | -0.16^***^ | -0.01 | -0.01 |
| Education^1^  High school or secondary school qualification | 0.06 | 0.08 | 0.03 | -0.11 | 0.22 | 0.06 | 0.08 | 0.03 | -0.09 | 0.22 |
| Education  Undergraduate/college degree level | 0.16 | 0.09 | 0.07 | -0.01 | 0.32 | 0.12 | 0.08 | 0.06 | -0.04 | 0.28 |
| Education  Graduate/Postgraduate degree level (e.g., Masters, PhD) | 0.45 | 0.10 | 0.15^***^ | 0.26 | 0.64 | 0.38 | 0.09 | 0.13^***^ | 0.20 | 0.56 |
| Gender^2^  Female | -0.15 | 0.04 | -0.07^***^ | -0.23 | -0.07 | -0.14 | 0.04 | -0.07^***^ | -0.22 | -0.06 |
| Survey Wave^3^  2023 | -0.13 | 0.04 | -0.06^**^ | -0.21 | -0.05 | -0.07 | 0.04 | -0.04 | -0.15 | 0.00 |
| Climate Concern | 0.32 | 0.02 | 0.29^***^ | 0.27 | 0.36 | 0.21 | 0.02 | 0.19^***^ | 0.17 | 0.25 |
| Mild climate anxiety | - | - | - | - | - | 0.41 | 0.05 | 0.20^***^ | 0.32 | 0.50 |
| High climate anxiety | - | - | - | - | - | 1.02 | 0.07 | 0.35^***^ | 0.88 | 1.15 |

^1^Reference for education is no formal education, ^2^Reference for gender is male, ^3^Reference for survey wave is 2022, ^**^ *p* <.01, ^***^ *p* <.001

**Table S3**

Total and Direct effects of Climate Concern on Likelihood of Engaging in Private-Sphere Behaviours: Heating

|  | *B* | *SEB* | *β* | 95% CI | | *B* | *SEB* | *β* | 95% CI | |
| --- | --- | --- | --- | --- | --- | --- | --- | --- | --- | --- |
|  |  |  |  | Lower | Upper |  |  |  | Lower | Upper |
|  | Total effect of climate concern | | | | | Direct effect of climate concern | | | | |
| Constant | 2.08 | 0.16 | - | 1.76 | 2.40 | 2.05 | 0.17 | - | 1.73 | 2.37 |
| Age | 0.01 | 0.00 | 0.07^**^ | 0.00 | 0.01 | 0.01 | 0.00 | 0.08^***^ | 0.00 | 0.01 |
| Education^1^  High school or secondary school qualification | 0.15 | 0.10 | 0.07 | -0.05 | 0.35 | 0.15 | 0.10 | 0.07 | -0.05 | 0.35 |
| Education  Undergraduate/college degree level | 0.21 | 0.10 | 0.09^*^ | 0.01 | 0.42 | 0.21 | 0.10 | 0.09^*^ | 0.01 | 0.41 |
| Education  Graduate/Postgraduate degree level (e.g., Masters, PhD) | 0.14 | 0.12 | 0.04 | -0.09 | 0.37 | 0.14 | 0.12 | 0.04 | -0.09 | 0.37 |
| Gender^2^  Female | 0.15 | 0.05 | 0.07^**^ | 0.05 | 0.25 | 0.15 | 0.05 | 0.07^**^ | 0.05 | 0.25 |
| Survey Wave^3^  2023 | -0.16 | 0.05 | -0.07^***^ | -0.26 | -0.07 | -0.16 | 0.05 | -0.07^**^ | -0.26 | -0.07 |
| Climate Concern | 0.37 | 0.03 | 0.31^***^ | 0.32 | 0.42 | 0.34 | 0.03 | 0.28^***^ | 0.29 | 0.40 |
| Mild climate anxiety | - | - | - | - | - | 0.16 | 0.06 | 0.07^**^ | 0.05 | 0.28 |
| High climate anxiety | - | - | - | - | - | 0.11 | 0.09 | 0.03 | -0.06 | 0.28 |

^1^Reference for education is no formal education, ^2^Reference for gender is male, ^3^Reference for survey wave is 2022, ^*^*p* <.05, ^**^ *p* <.01, ^***^ *p* <.001

**Table S4**

Total and Direct effects of Climate Concern on Likelihood of Engaging in Private-Sphere Behaviours: Material Consumption

|  | *B* | *SEB* | *β* | 95% CI | | *B* | *SEB* | *β* | 95% CI | |
| --- | --- | --- | --- | --- | --- | --- | --- | --- | --- | --- |
|  |  |  |  | Lower | Upper |  |  |  | Lower | Upper |
|  | Total effect of climate concern | | | | | Direct effect of climate concern | | | | |
| Constant | 2.43 | 0.12 | - | 2.18 | 2.67 | 2.25 | 0.12 | - | 2.01 | 2.48 |
| Age | -0.01 | 0.00 | -0.26^***^ | -0.02 | -0.01 | -0.01 | 0.00 | -0.19^***^ | -0.01 | -0.01 |
| Education^1^  High school or secondary school qualification | 0.03 | 0.08 | 0.02 | -0.12 | 0.18 | 0.04 | 0.07 | 0.02 | -0.11 | 0.18 |
| Education  Undergraduate/college degree level | 0.13 | 0.08 | 0.06 | -0.02 | 0.28 | 0.11 | 0.08 | 0.05 | -0.04 | 0.26 |
| Education  Graduate/Postgraduate degree level (e.g., Masters, PhD) | 0.27 | 0.09 | 0.10^**^ | 0.10 | 0.44 | 0.23 | 0.09 | 0.08^**^ | 0.06 | 0.39 |
| Gender^2^  Female | 0.19 | 0.04 | 0.10^***^ | 0.11 | 0.26 | 0.19 | 0.04 | 0.10^***^ | 0.12 | 0.27 |
| Survey Wave^3^  2023 | -0.03 | 0.04 | -0.02 | -0.11 | 0.04 | 0.00 | 0.04 | 0.00 | -0.07 | 0.07 |
| Climate Concern | 0.34 | 0.02 | 0.33^***^ | 0.30 | 0.38 | 0.25 | 0.02 | 0.25^***^ | 0.21 | 0.29 |
| Mild climate anxiety | - | - | - | - | - | 0.39 | 0.04 | 0.21^***^ | 0.31 | 0.48 |
| High climate anxiety | - | - | - | - | - | 0.70 | 0.06 | 0.25^***^ | 0.57 | 0.82 |

^1^Reference for education is no formal education, ^2^Reference for gender is male, ^3^Reference for survey wave is 2022, ^*^*p* <.05, ^**^ *p* <.01, ^***^ *p* <.001

**Table S5**

Total and Direct effects of Climate Concern on Likelihood of Engaging in Climate Activism: Non-Violent Disruptive Protests

|  | *B* | *SEB* | *β* | 95% CI | | *B* | *SEB* | *β* | 95% CI | |
| --- | --- | --- | --- | --- | --- | --- | --- | --- | --- | --- |
|  |  |  |  | Lower | Upper |  |  |  | Lower | Upper |
|  | Total effect of climate concern | | | | | Direct effect of climate concern | | | | |
| Constant | 1.46 | 0.18 | - | 1.10 | 1.81 | 1.14 | 0.17 | - | 0.80 | 1.49 |
| Age | -0.02 | 0.00 | -0.24^***^ | -0.02 | -0.02 | -0.01 | 0.00 | -0.17^***^ | -0.02 | -0.01 |
| Education^1^  High school or secondary school qualification | 0.14 | 0.11 | 0.05 | -0.08 | 0.36 | 0.15 | 0.11 | 0.06 | -0.06 | 0.36 |
| Education  Undergraduate/college degree level | 0.31 | 0.11 | 0.10^**^ | 0.08 | 0.53 | 0.28 | 0.11 | 0.10^*^ | 0.06 | 0.49 |
| Education  Graduate/Postgraduate degree level (e.g., Masters, PhD) | 0.40 | 0.13 | 0.10^**^ | 0.15 | 0.65 | 0.33 | 0.12 | 0.08^**^ | 0.09 | 0.57 |
| Gender^2^  Female | 0.05 | 0.06 | 0.02 | -0.06 | 0.16 | 0.06 | 0.05 | 0.02 | -0.04 | 0.17 |
| Survey Wave^3^  2023 | -0.02 | 0.06 | -0.01 | -0.13 | 0.09 | 0.04 | 0.05 | 0.02 | -0.06 | 0.15 |
| Climate Concern | 0.55 | 0.03 | 0.38^***^ | 0.49 | 0.60 | 0.40 | 0.03 | 0.27^***^ | 0.34 | 0.46 |
| Mild climate anxiety | - | - | - | - | - | 0.62 | 0.06 | 0.23^***^ | 0.50 | 0.74 |
| High climate anxiety | - | - | - | - | - | 1.20 | 0.09 | 0.30^***^ | 1.02 | 1.38 |

^1^Reference for education is no formal education, ^2^Reference for gender is male, ^3^Reference for survey wave is 2022, ^***^ *p* <.001, ^**^ *p* <.01, ^*^ *p* <.05

**Table S6**

Total and Direct effects of Climate Concern on Likelihood of Engaging in Climate Activism: Communicating with Friends and Relatives

|  | *B* | *SEB* | *β* | 95% CI | | *B* | *SEB* | *β* | 95% CI | |
| --- | --- | --- | --- | --- | --- | --- | --- | --- | --- | --- |
|  |  |  |  | Lower | Upper |  |  |  | Lower | Upper |
|  | Total effect of climate concern | | | | | Direct effect of climate concern | | | | |
| Constant | 1.43 | 0.16 | - | 1.12 | 1.75 | 1.15 | 0.15 | - | 0.85 | 1.45 |
| Age | -0.01 | 0.00 | -0.19^***^ | -0.02 | -0.01 | -0.01 | 0.00 | -0.11^***^ | -0.01 | -0.01 |
| Education^1^  High school or secondary school qualification | 0.16 | 0.10 | 0.07 | -0.03 | 0.36 | 0.18 | 0.09 | 0.07 | -0.01 | 0.36 |
| Education  Undergraduate/college degree level | 0.38 | 0.10 | 0.14^***^ | 0.18 | 0.58 | 0.34 | 0.10 | 0.13^***^ | 0.16 | 0.53 |
| Education  Graduate/Postgraduate degree level (e.g. Masters, PhD) | 0.40 | 0.11 | 0.11^***^ | 0.17 | 0.62 | 0.34 | 0.11 | 0.09^**^ | 0.13 | 0.55 |
| Gender^2^  Female | 0.04 | 0.05 | 0.02 | -0.06 | 0.14 | 0.05 | 0.05 | 0.02 | -0.05 | 0.14 |
| Survey Wave^3^  2023 | -0.11 | 0.05 | -0.04^*^ | -0.20 | -0.01 | -0.05 | 0.05 | -0.02 | -0.14 | 0.04 |
| Climate Concern | 0.53 | 0.03 | 0.41^***^ | 0.48 | 0.58 | 0.39 | 0.03 | 0.30^***^ | 0.34 | 0.44 |
| Mild climate anxiety | - | - | - | - | - | 0.61 | 0.05 | 0.25^***^ | 0.50 | 0.71 |
| High climate anxiety | - | - | - | - | - | 1.10 | 0.08 | 0.31^***^ | 0.95 | 1.26 |

^1^Reference for education is no formal education, ^2^Reference for gender is male, ^3^Reference for survey wave is 2022, ^***^ *p* <.001, ^**^ *p* <.01, ^*^ *p < .05*

**Table S7**

Total and Direct effects of Climate Concern on Likelihood of Engaging in Climate Activism: Community Action

|  | *B* | *SEB* | *β* | 95% CI | | *B* | *SEB* | *β* | 95% CI | |
| --- | --- | --- | --- | --- | --- | --- | --- | --- | --- | --- |
|  |  |  |  | Lower | Upper |  |  |  | Lower | Upper |
|  | Total effect of climate concern | | | | | Direct effect of climate concern | | | | |
| Constant | 1.67 | 0.17 | - | 1.34 | 2.01 | 1.31 | 0.16 | - | 1.00 | 1.63 |
| Age | -0.02 | 0.00 | -0.24^***^ | -0.02 | -0.02 | -0.01 | 0.00 | -0.14^***^ | -0.01 | -0.01 |
| Education^1^  High school or secondary school qualification | 0.10 | 0.11 | 0.04 | -0.11 | 0.31 | 0.11 | 0.10 | 0.04 | -0.08 | 0.30 |
| Education  Undergraduate/college degree level | 0.27 | 0.11 | 0.10^*^ | 0.06 | 0.48 | 0.22 | 0.10 | 0.08^*^ | 0.03 | 0.42 |
| Education  Graduate/Postgraduate degree level (e.g., Masters, PhD) | 0.53 | 0.12 | 0.14^***^ | 0.29 | 0.77 | 0.44 | 0.11 | 0.12^***^ | 0.22 | 0.66 |
| Gender^2^  Female | 0.01 | 0.05 | 0.01 | -0.09 | 0.12 | 0.03 | 0.05 | 0.01 | -0.07 | 0.13 |
| Survey Wave^3^  2023 | -0.04 | 0.05 | -0.02 | -0.14 | 0.06 | 0.03 | 0.05 | 0.01 | -0.06 | 0.13 |
| Climate Concern | 0.47 | 0.03 | 0.35^***^ | 0.42 | 0.53 | 0.30 | 0.03 | 0.22^***^ | 0.25 | 0.36 |
| Mild climate anxiety | - | - | - | - | - | 0.69 | 0.06 | 0.27^***^ | 0.58 | 0.80 |
| High climate anxiety | - | - | - | - | - | 1.43 | 0.08 | 0.39^***^ | 1.27 | 1.59 |

^1^Reference for education is no formal education, ^2^Reference for gender is male, ^3^Reference for survey wave is 2022, *^***^ p <.001, ^*^ p < .05.*
